# Supplementary material for: Recent Advances in the Use of Molecular Methods for the Diagnosis of Bacterial Infections
Source: Pathogens. 2022 Jun 8;11(6):663. doi: 10.3390/pathogens11060663 (PMC9229729; doi:10.3390/pathogens11060663)
Supplement: Supplementary file 1 [file pathogens-11-00663-s001.zip › pathogens-1725935-supplementary.pdf]

Table S1. Summary of available technologies.

Methods	**References**
PCR	[92,93]
Real-Time PCR	[36,94]
LAMP	[95,96]
NASBA	[97,98]
TMA	[98,99]
HDA	[49,100]
The BioFire FilmArray panels	[101,102]
DNA microarrays	[60,61]
Pulse-Field Gel Electrophoresis (PFGE)	[68,103]
Multilocus Sequencing Typing (MLST)	[104,105]
Whole Genome Sequencing (WGS)	[71,106]
Pyrosequencing	[70,107]
Next-Generation Sequencing (NGS)	[73,108]
Microfluidics	[75,76]
Immunodetection of Pathogens	[28,109]
Detection of Growth-Related Molecules	[28,77,78]
Biosensor Systems	[80–82]
